# Evaluation of the Efficacy and Safety of Transjugular Intrahepatic Portosystemic Shunt Combined With Concurrent Antegrade Embolization of Large Spontaneous Portosystemic Shunts

**DOI:** 10.1111/1754-9485.13832

**Published:** 2025-02-21

**Authors:** Ze Wang, Xiao‐Yang Xu, Chen‐You Liu, Jin‐Tao Huang, Wan‐Ci Li, Shuai Zhang, Jian Shen, Bin‐Yan Zhong, Xiao‐Li Zhu

**Affiliations:** ^1^ Department of Interventional Radiology The First Affiliated Hospital of Soochow University Suzhou People's Republic of China

**Keywords:** large spontaneous portosystemic shunts, oesophagogastric variceal bleeding, portal hypertension, TIPS

## Abstract

**Objectives:**

To compare the long‐term efficacy and safety of transjugular intrahepatic portosystemic shunt (TIPS) combined with concurrent antegrade embolization in treating portal hypertension with oesophagogastric variceal bleeding in patients with and without large spontaneous portosystemic shunts (L‐SPSSs).

**Materials and Methods:**

We retrospectively analysed data from patients with portal hypertension who underwent TIPS from November 2015 to April 2022. The patients were screened according to the inclusion criteria and were divided into L‐SPSSs group (L‐S group) and Non L‐SPSSs group (Non L‐S group). The primary outcome was the 2‐year liver transplantation‐free survival (TFS) rate. Secondary outcomes contained the incidence of overt hepatic encephalopathy (OHE), ectopic embolization and the 2‐year rebleeding rate.

**Results:**

A total of 259 patients were enrolled (64 patients in L‐S group and 195 patients in Non L‐S group). The average age was 57.2 years, and the success rate of procedure was 100%. Baseline data showed no significant differences between two groups. There was a statistically significant difference in the 2‐year liver transplantation‐free rate between two groups (L‐S vs. Non L‐S, 84.38% vs. 71.28%; *p* = 0.045). OHE occurred in 19 (29.69%) patients with L‐SPSSs and 104 (53.33%) patients without L‐SPSSs, with a statistically significant difference (*p* = 0.001). And no statistically significant difference was found in ectopic embolism incidence rate and the 2‐year rebleeding rate between two groups. Multivariate Cox regression analysis identified male gender, portal vein thrombosis and preoperative high blood ammonia levels as independent risk factors for long‐term survival.

**Conclusion:**

Compared to Non L‐S group, the patients in L‐S group achieve longer liver transplantation‐free survival and lower incidence rate of OHE without increasing the risk of 2‐year rebleeding and ectopic embolization.

Liver cirrhosis can lead to various kinds of complications, including ascites, variceal bleeding caused by portal hypertension (PH) and the development of hepatic encephalopathy (HE) [1]. To decompress the portal vein system, spontaneous venous collateral circulation forms, allowing blood to bypass the liver and divert to the systemic venous system through spontaneous portosystemic shunts (SPSSs) [2].

These shunts are common in liver cirrhosis, occurring in up to 60% of patients, with half having large diameter SPSS (A large SPSS is defined as an SPSS diameter greater than 8 mm or a sum of SPSS diameters greater than half the diameter of the main portal vein [3]), known as large spontaneous portosystemic shunts (L‐SPSSs), including splenorenal shunts (SRSs), gastrorenal shunts (GRSs), paraumbilical veins (PUVs), portocaval or mesorenal/caval shunts, gastrocaval shunts (ECSs), and gastric varices (GVs). Such collateral circulations result from portal hypertension, creating an inflammatory and angiogenic environment. Their presence is often associated with clinically portal hypertension [4, 5]. Large shunts may be a maladaptive phenomenon and are often associated with persistent, recurrent, or refractory hepatic encephalopathy (HE). Embolizing L‐SPSSs can improve hepatic perfusion, HE, liver fibrosis, liver function, and overall survival. Although SPSSs were traditionally viewed as a compensatory mechanism for portal hypertension, recent evidence suggests they do not adequately reduce portal pressure and may decrease blood perfusion. The Baveno VII collaboration found that SPSSs appeared in up to 55% of patients with compensated cirrhosis, with a total surface area > 83 mm^2^ predictive of overt HE and mortality [6].

Thus, selective embolization of L‐SPSSs appears promising. A retrospective study showed that SPSSs ≥ 6 mm were associated with overt HE, and embolization reduced overt HE risk by nearly 50% without affecting mortality, shunt dysfunction, or clinical recurrence [7]. Similar findings by Leng et al. indicated that combining TIPS with shunt embolization is a superior treatment for variceal bleeding [8]. Large SPPSs used to be occluded in a retrograde method, while TIPS combined with antegrade embolization was performed as a routine procedure for small variceal spontaneous portosystemic shunts. However, there is a lack of studies on proving the efficacy of concurrent antegrade embolization of large shunts.

In order to solve the risks of large spontaneous portosystemic shunts, there is yet to be a clear consensus on whether balloon retrograde transvenous obliteration (BRTO) or TIPS is the preferred option for managing GVs. In these studies, analytic statistics were lacking on prognosis evaluation and intraoperative period complications, such as ectopic embolization, puncture tract bleeding and so on. Therefore, our study records the incident of ectopic embolism during concurrent antegrade L‐SPSSs embolization during TIPS creation.

The purpose of this study was to demonstrate the efficacy and safety in patients with cirrhotic portal hypertension and variceal bleeding in the presence of L‐SPSSs who underwent concurrent antegrade L‐SPSSs embolization during TIPS creation.

## 1 Materials and Methods

### 1.1 General Information

From November 2015 to April 2022, patients with cirrhotic portal hypertension and variceal bleeding who underwent TIPS at the First Affiliated Hospital of Soochow University were retrospectively screened. Inclusion criteria were: (1) Definitive diagnosis of liver cirrhosis (based on clinical manifestations, laboratory tests, imaging or liver biopsy); (2) Diagnosis of oesophageal and gastric variceal bleeding (EGVB) based on medical history, clinical manifestations, and laboratory examination, with oesophageal and gastric varices (EGVs) confirmed by enhanced CT or endoscopy. (3) Child‐Pugh score < 14; (4) Compliance with Baveno VII guidelines for TIPS indications. Exclusion criteria included: (1) Other TIPS indications such as refractory ascites; (2) Previous surgical shunt or TIPS procedure; (3) Preoperative liver malignancy or other malignancies; (4) hepatic encephalopathy prior; (5) Loss of follow‐up. Patients were divided into two groups based on the presence or absence of L‐SPSSs: 64 cases in the L‐SPSSs group and 195 cases in the Non L‐SPSSs group.

### 1.2 Preoperative Evaluation

Preoperative contrast‐enhanced abdominal CT scans were performed using an Evolution 256‐row CT scanner. A total of 80–100 mL of ioversol (320mgI/ml) was injected through the median cubital vein with a high‐pressure syringe at a flow rate of 2.0–3.0 mL/s, Portal phase images were acquired 45–50 s after contrast agent injection. Scanning parameters were tube voltage 120 kV, tube current 200–450 mA/s, slice thickness 5 mm, pitch 0.992, and X‐ray tube rotation speed 0.5 s/cycle. The reconstruction slice thickness and interval was 1.25 mm. Portal venous phase images were processed using the GE ADW4.7 image post‐processing workstation, using multi planar reconstruction (MPR), maximum intensity projection (MIP), and volume reconstruction (VR) techniques to reconstruct the portal vein system and its collateral vessels, plan the surgical puncture path, and evaluate the presence of SPSSs.

### 1.3 Treatments and Follow Up

TIPS procedures were performed by a team leader with over 10 years of experience interventional radiologists. Using a transjugular approach, a standard TIPS device (RUPS‐100; Cook Medical) was advanced to the hepatic vein, followed by puncturing the portal vein. Successful portal vein puncture was confirmed by portography, after which concurrent anterograde embolization was performed using coils (Cook, Bloomington, IN, USA) and/or tissue gel (BME, Guangzhou, China). The parenchymal tract was then dilated with a 6 or 8 mm‐diameter balloon (Boston Scientific, Marlborough, Massachusetts, USA, or Abbott, Chicago, IL, USA) which was then selected to dilate the shunt until the incision had completely disappeared, and an 8 mm diameter Viatorr stent (W. L. Gore & Associates Inc., Newark, USA) was implanted. The patients receiving TIPS underwent routine physical examinations, laboratory tests, abdominal ultrasound, and contrast‐enhanced CT before the procedure and at 3 days, 1 month, and 3 months post‐procedure. Subsequent follow‐up was conducted at 6‐month intervals. In case of upper gastrointestinal bleeding (UGIB) or other serious complications, patients were instructed to return to the hospitals. Follow‐up was completed on 30 April 2024.

### 1.4 Outcomes and Definitions

The primary outcome was 2‐year transplantation‐free survival. Secondary outcomes included the 2‐year rebleeding rate, incidence of overt hepatic encephalopathy (OHE), and stent dysfunction. OHE was defined and graded according to the Westport criteria [9], with Grade 2 and above considered OHE. Rebleeding was defined as the occurrence of persistent or new clinical bleeding symptoms per the Baveno VII consensus. Stent dysfunction was identified by a flow velocity in the stent of less than 40 cm/s or greater than 200 cm/s, low portal vein flow velocity (< 20 cm/s) on ultrasound, or shunt stenosis exceeding 50% [10].

### 1.5 Research Methods

Statistical analysis was conducted using SPSS version 26.0 (IBM, New York, USA). Continuous variables were expressed as median and interquartile range for non‐normal data and compared using the Mann–Whitney *U*‐test. Normally distributed data were expressed as mean ± standard deviation and analysed using the *t*‐test. Univariate and multivariate regression analyses (including COX and logistic) identified risk factors. Kaplan–Meier method was used for survival analysis, with *p* < 0.05 considered statistically significant. The surv_cutpoint function in the survminer package of R language was used for hierarchical analysis and to determine the optimal cut‐off value for continuous variables and draw survivorship curve.

## 2 Results

### 2.1 Study Patients

**FIGURE 1:**
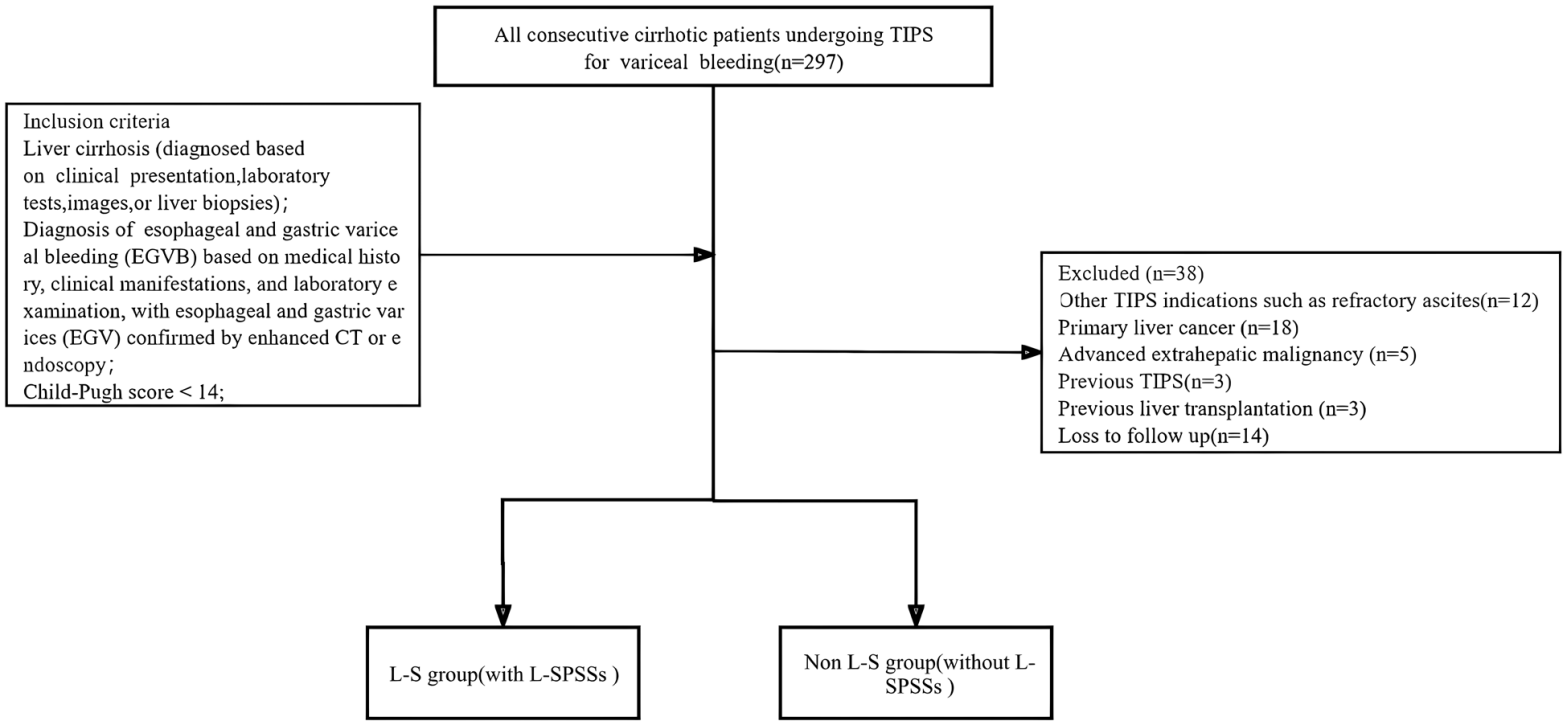
Flowchart showing the study design and patient disposition.

Of 297 patients underwent TIPS, 38 were excluded for various reasons (Figure 1), leaving 259 patients (160 males and 99 females) aged from 26 to 83 years, with an average age of 57.2 years. All patients had chronic liver disease and portal hypertension, presenting with decompensated liver cirrhosis and acute variceal bleeding. None had prior TIPS treatment. Among these, 64 patients with L‐SPSSs received TIPS combined with L‐SPSSs antegrade embolization (L‐S group, *n* = 64), and 195 patients who didn't have large spontaneous portosystemic shunts and variceal but had small spontaneous portosystemic shunts received TIPS combined with antegrade embolization (Non L‐S group, *n* = 195). Baseline characteristics were comparable between the groups (Table 1). All patients completed the TIPS procedure successfully with complete embolization using coils or tissue gel achieved in all patients, without serious embolism‐related complications. All patients reached the end point of follow‐up.

**TABLE 1:** Baseline characteristics of patients.

Variable	Non L‐S (*n* = 195)	L‐S (*n* = 64)	*p*
Age (years)	60.00 [50.00, 67.00]	55.00 [47.75, 65.00]	0.173
Male gender			
Male	118 (60.51)	42 (65.62)	0.465
Female	77 (39.49)	22 (34.38)	
Aetiology of cirrhosis			
Hepatitis virus infection	94 (48.21)	37 (57.81)	0.182
Non hepatitis virus infection	101 (51.79)	27 (42.19)	
Child‐Pugh score(points)	7.00 [6.00, 9.00]	7.00 [6.00, 8.00]	0.056
Child‐Pugh class			
A	65 (33.33)	31 (48.44)	0.065
B	111 (56.92)	26 (40.62)	
C	19 (9.74)	7 (10.94)	
MELD score(points)	10.00 [9.00, 13.00]	11.00 [8.00, 13.00]	0.763
Type of GOV			
GOV1	152 (77.9)	51 (79.6)	0.874
GOV2	37 (19.0)	11 (17.2)	
IGV1	6 (3.1)	2 (3.2)	
PVT			
Without	149 (76.41)	48 (75.00)	0.819
With	46 (23.59)	16 (25.00)	
Laboratory tests			
TBIL (μmol/L)	20.70 [14.65, 29.05]	20.25 [15.12, 27.23]	0.41
Cr (μmol/L)	62.30 [53.00, 75.55]	62.95 [48.53, 68.30]	0.196
BUN (mmol/L)	7.80 [5.60, 10.18]	6.55 [5.40, 9.33]	0.111
ALB(g/L)	31.12 (5.63)	31.49 (6.16)	0.661
PT(s)	15.80 [14.40, 17.10]	16.10 [14.38, 18.75]	0.222
AST(U/L)	32.30 [23.75, 48.40]	30.25 [23.48, 42.25]	0.385
ALT(U/L)	28.00 [19.00, 45.00]	26.15 [14.52, 38.17]	0.11
PLT(×10^9^/L)	73.00[52.00, 106.00]	67.50 [48.25, 89.75]	0.225
WBC (×10^9^/L)	5.34 [3.12, 8.41]	5.04 [3.72, 7.77]	0.901
INR	1.33 [1.21, 1.46]	1.38 [1.23, 1.58]	0.208
NH3 (μmol/L)	30.20 [16.90, 46.05]	29.40 [18.92, 45.50]	0.857

*Note:* Results are expressed as the number of patients (%) or median with interquartile range.

Abbreviations: ALB, albumin; ALT, alanine transaminase; AST, aspartate aminotransferase; BUN, blood urea nitrogen; Cr, creatinine; GOV, gastroesophageal varices; INR, international normalised ratio; MELD, Model for End‐Stage Liver Disease; PLT, platelet count; TBIL, total bilirubin; WBC, white blood cells.

### 2.2 Primary Outcome

**FIGURE 2:**
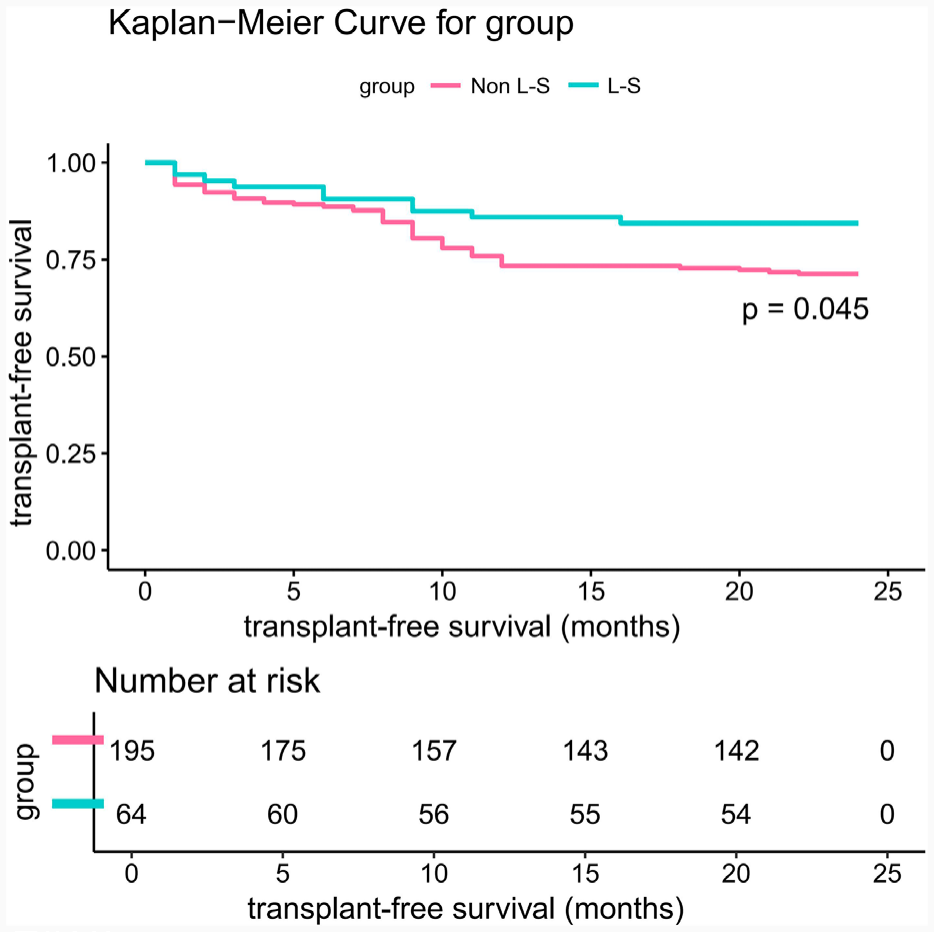
Analyses of the primary outcome of 2‐year transplantation‐free survival. Kaplan–Meier curves showing the cumulative incidence of 2‐year transplantation‐free survival rate after randomization. Censoring of the data is indicated by the vertical bars. Data were censored on the date of endpoint, at the time of death, at the time of the last documented visit, or at the end of the complete follow‐up period, whichever occurred earliest.

**FIGURE 3:**
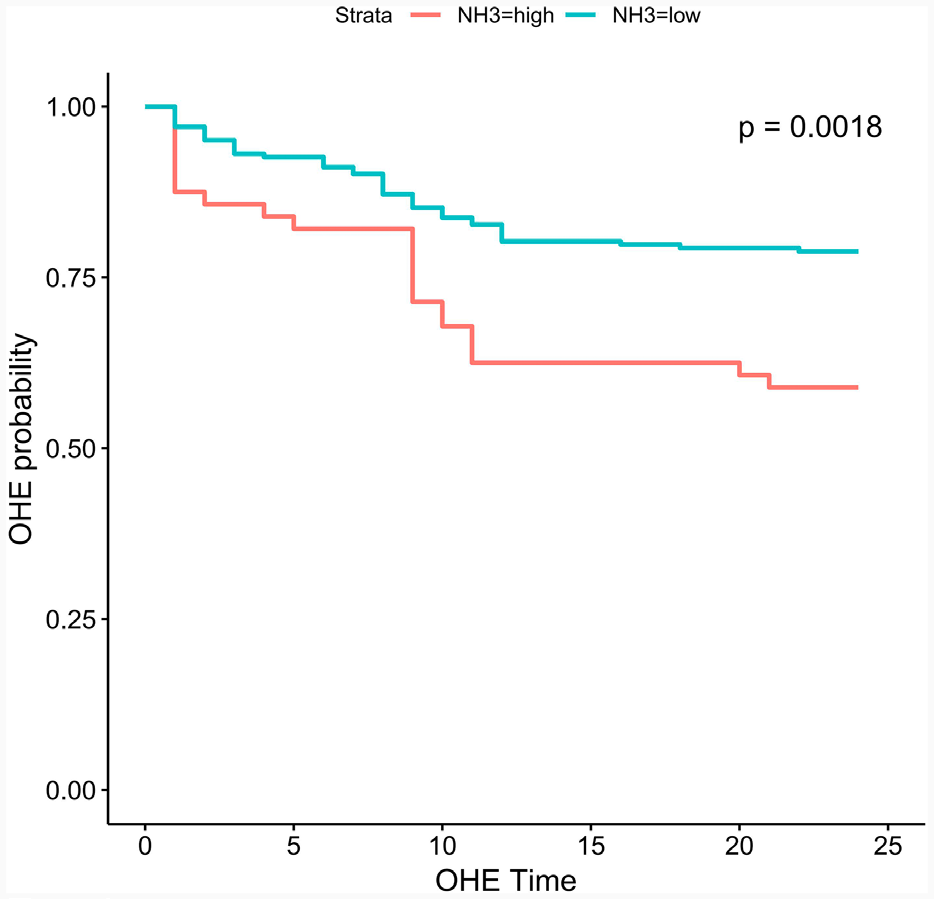
Based on the cut‐off value of 50 μmol/L, analyses of the outcome of 2‐year transplantation‐free survival. Kaplan–Meier curves showing the cumulative incidence of 2‐year transplantation‐free survival rate after randomization.

**FIGURE 4:**
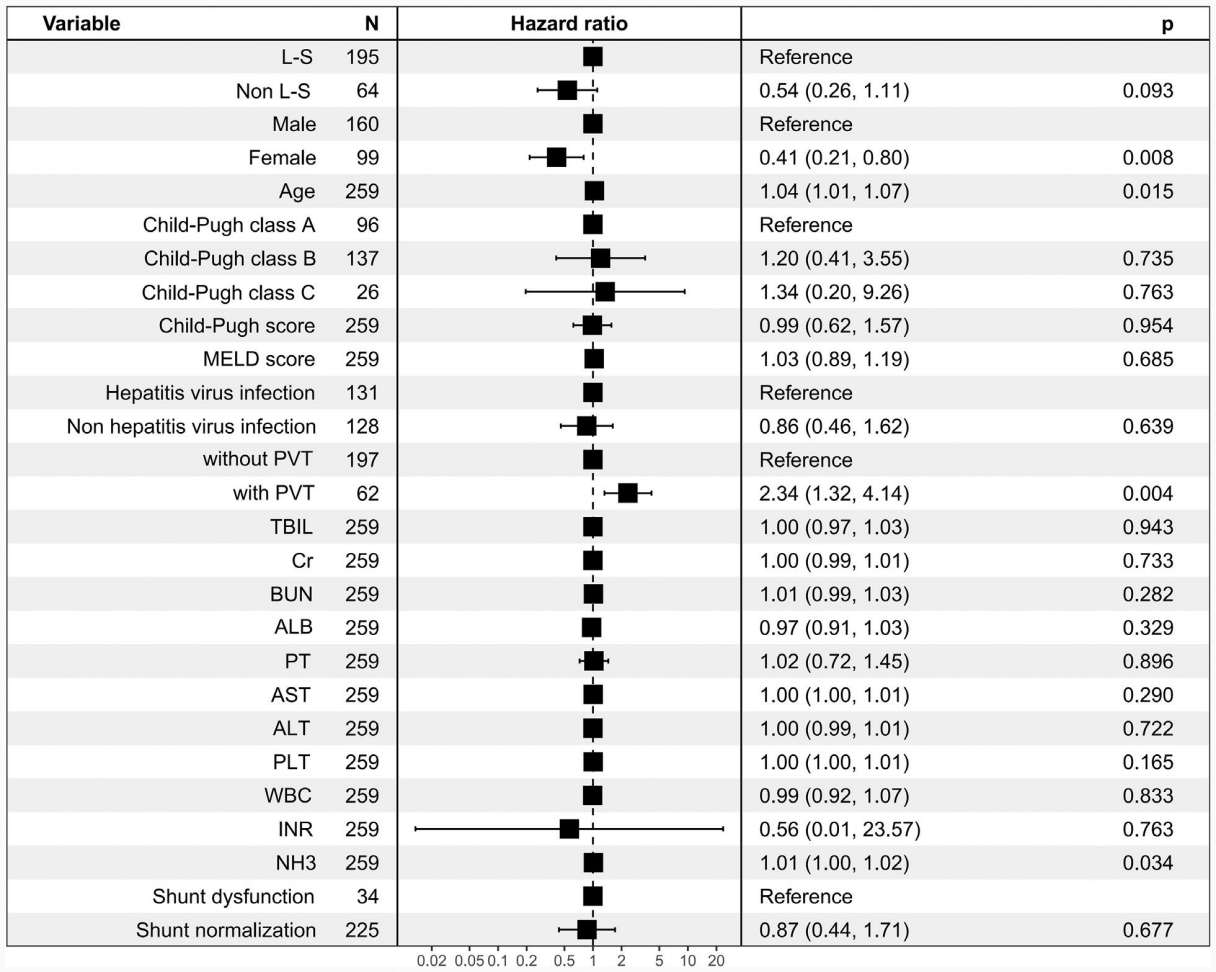
Forest plots showing the confounder‐adjusted effecis of L‐SPSSs versus Non L‐S on the development of 2‐year transplantation‐free survival rate after TIPS. HRs are derived from multivariable Cox regression models, variables with p < 0.10 in univariable analyses were included in the multivariable analysis.

The 2‐year liver transplantation‐free survival rate significantly differed between the groups (L‐S vs. Non L‐S, 84.38% vs. 71.28%; HR, 0.51; 95% CI, 0.26–1.00; *p* = 0.045, Figure 2). In the Non L‐S group, 18 patients (9.23%) died within 3 month postoperatively, with 13 dying of uncontrolled variceal bleeding and five of acute liver failure. In the L‐S group, four patients (6.25%) died within 3 months, with three dying of uncontrolled variceal bleeding and one of acute liver failure. There was no significant difference in three‐month mortality rates between the groups (L‐S vs. Non L‐S, 6.25% vs. 9.23%, *p* = 0.458). Blood ammonia levels were stratified using the surv_cutpoint function in the R survminer package, with a cut‐off value of 50 μmol/L. Patients with high blood ammonia had significantly lower 2‐year transplantation‐free survival rates than those with lower levels (Figure 3). Univariate and multivariate regression analyses (including COX and logistic) identified male, portal vein thrombosis, and high blood ammonia as independent risk factors for postoperative survival (Figure 4).

### 2.3 Secondary Outcomes

**FIGURE 5:**
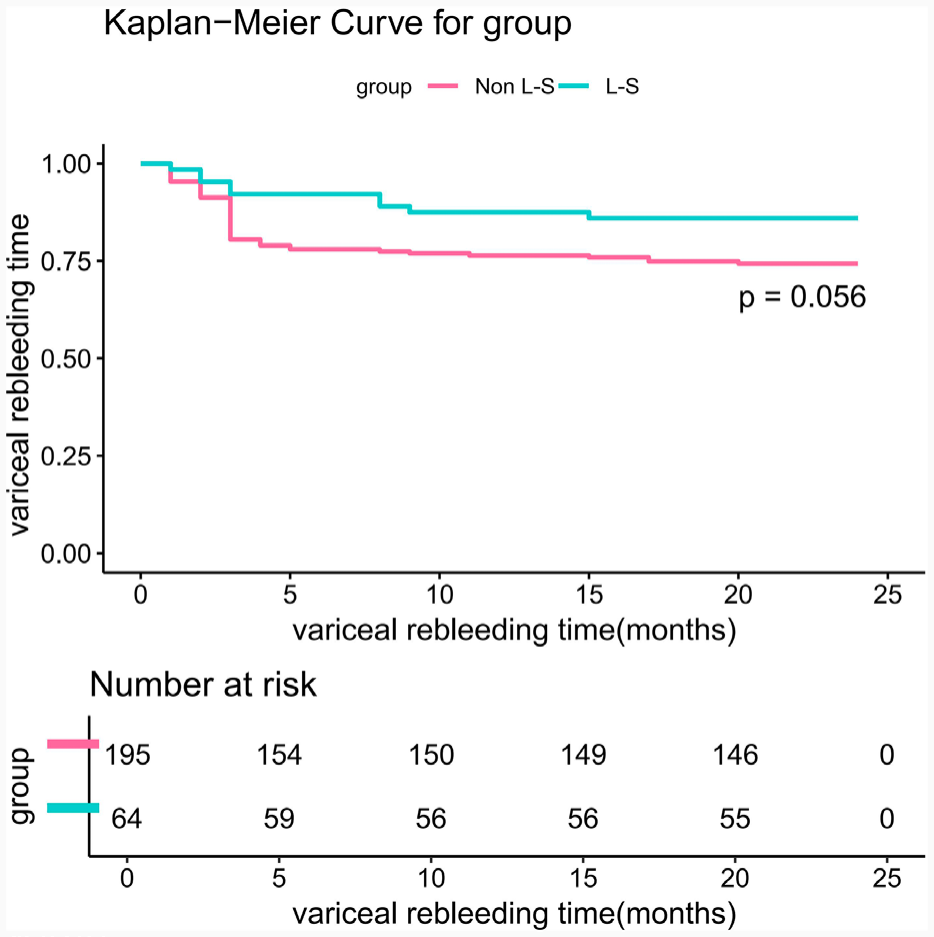
Analyses of the secondary outcome of 2‐year rebleeding. Kaplan–Meier curves showing the cumulative incidence of 2‐year transplantation‐free survival rate after randomization. Censoring of the data is indicated by the vertical bars. Data were censored on the date of endpoint, at the time of death, at the time of the last documented visit, or at the end of the complete follow‐up period, whichever occurred earliest.

**FIGURE 6:**
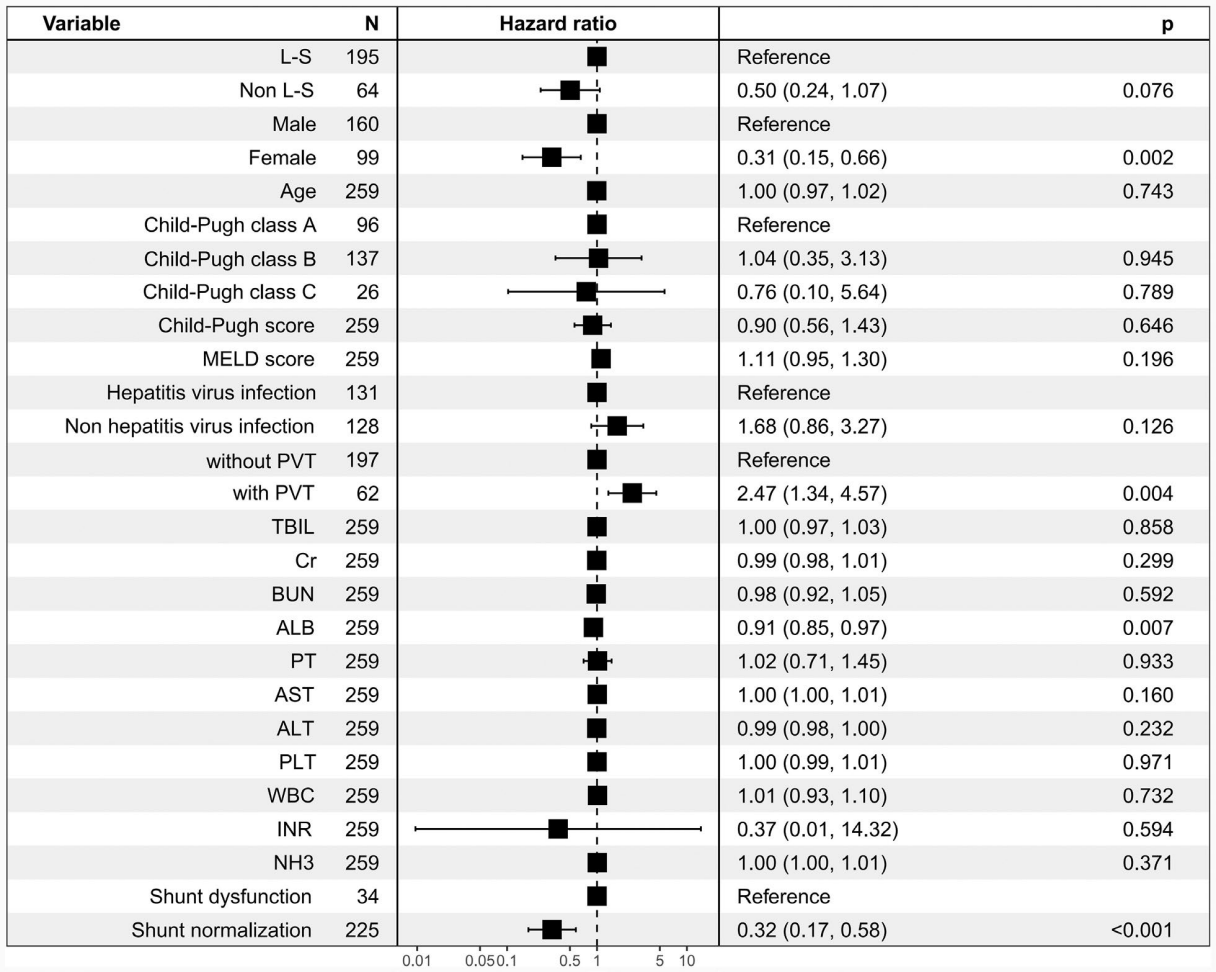
Forest plots showing the confounder‐adjusted effecis of L‐S group versus Non L‐S group on the development of 2‐year rebleeding rate after TIPS. HRs are derived from multivariable Cox regression models, Variables with *p* < 0.10 in univariable analyses were included in the multivariable analysis.

There was no significant difference in the 2‐year rebleeding rate between the groups (L‐S vs. Non L‐S, 14.06% vs. 25.64%; HR, 0.51; 95% CI, 0.25–1.03; *p* = 0.056, Figure 5). In the Non L‐S group, 38 patients (19.49%) rebleeded within 3 months postoperatively; all received conservative treatment, with 13 dying of uncontrolled variceal rebleeding. The remaining patients' haemorrhage was controlled after conservative treatment. In the L‐S group, five patients (7.81%) experienced bleeding within 3 months postoperatively; all received conservative treatment, with three dying of uncontrolled variceal bleeding and two undergoing successful shunt revision with no further again. There was a statistically significant difference in the 3‐month rebleeding rates between the groups (L‐S vs. Non L‐S, 7.81% vs. 19.49%, *p* = 0.016). Univariate and multivariate regression analyses (including COX and logistic) identified male gender, portal vein thrombosis, and low albumin levels as independent risk factors for postoperative rebleeding (Figure 6).

And no statistically significant difference was found in ectopic embolism incidence rate between two groups (L‐S vs. Non L‐S, 3.12% vs. 5.64%, *p* = 0.424). In L‐S group, two patients both occurred pulmonary embolization. In Non L‐S group, one patient occurred cerebral embolization and nine patients occurred pulmonary embolization.

There was a significant difference in the incidence of OHE between the groups (L‐S vs. Non L‐S, 29.69% vs. 53.33%, *p* < 0.001). All OHE patients were hospitalised and received treatment with lactulose combined with rifaximin or ornithine aspartate.

## 3 Discussion

This retrospective controlled study demonstrates that TIPS combined with concurrent antegrade embolization of L‐SPSSs offers superior long‐term outcomes for cirrhotic patients with portal hypertension and variceal bleeding. Meanwhile, combined concurrent antegrade embolization will not increase the risk of intraoperative ectopic embolization. The management of oesophagogastric variceal bleeding with L‐SPSSs often involves minimally invasive endovascular treatments BRTO and TIPS. However, the efficacy and outcomes of these treatments can vary depending on individual and institutional factors. Therefore, there is yet to be a clear consensus on whether BRTO or TIPS is the preferred option. Our study supports that: TIPS combined with concurrent anterograde embolization has emerged as a promising treatment option for oesophagogastric variceal bleeding. Key findings include improved long‐term survival and a reduced incidence of overt hepatic encephalopathy (HE). These results underscore several critical aspects of managing these patients and highlight important considerations for clinical practice. Our study indicates that patients undergoing TIPS combined with L‐SPSSs concurrent anterograde embolization have a better long‐term survival prognosis. The data suggest that male gender, portal vein thrombosis, and elevated blood ammonia levels are independent risk factors for poorer postoperative survival. Notably, we identified an optimal cut‐off value of 50 μmol/L for blood ammonia levels, above which the risk of mortality significantly increases. This stratification could be valuable for identifying high‐risk patients who might benefit from more intensive monitoring and management. A significant finding is the reduction in the incidence of overt HE among patients receiving combined TIPS and L‐SPSSs concurrent anterograde embolization. In our study, the L‐S group had an overt HE incidence of 29.69%, significantly lower than the 53.33% in the Non L‐S group. This reduction can be attributed to decreased systemic exposure to gut‐derived toxins, such as ammonia, which bypass hepatic detoxification due to portosystemic shunts. Concurrent anterograde embolizing L‐SPSSs likely mitigates this risk by increasing hepatic blood flow and improving detoxification processes, thereby enhancing patient outcomes and quality of life. Bizarrely, while TIPS combined with L‐SPSSs concurrent anterograde embolization did not significantly reduce the long‐term rebleeding risk, it did lower the short‐term (within 3 months) rebleeding rates. This discrepancy might be due to hemodynamic changes post‐embolization that improve stent patency rates and overall portal hemodynamics, thus contributing to better short‐term outcomes. Moreover, the combined procedure did not increase the risk of stent dysfunction or other complications, which is critical for maintaining patient safety and procedural efficacy. Randomised controlled trials (RCTs) by Guohong Han et al. have supported similar findings, indicating no significant difference in long‐term rebleeding rates between combined treatment and TIPS alone, despite short‐term improvements. These findings align with North American practice‐based recommendations for TIPS, which suggest considering embolization of SPSSs > 6 mm to reduce the risk of overt HE after TIPS [11]. In this study, the incidence of overt HE was 53.33% in the Non L‐S group, confirming a higher risk in this cohort [7]. In contrast, the incidence of overt HE dropped to 29.69% in the L‐S group, approximately half that of the Non L‐S group. These results support that joint concurrent anterograde embolization of L‐SPSSs during TIPS can effectively reduce the risk of postoperative HE. The correlation between portosystemic shunts and HE has been established for decades, applicable to both spontaneous and iatrogenic shunts (e.g., TIPS and surgical shunts). In both scenarios, significant volumes of venous blood bypass the liver, leading to systemic circulation of toxic compounds, primarily from the gut. These compounds, such as ammonia, which would typically be detoxified by the liver, accumulate in the plasma, resulting in neuropsychiatric symptoms that impair patient autonomy, awareness, behaviour, and mental functioning [2, 12]. Embolization of L‐SPSSs reduces the volume of shunted blood, thereby lowering brain exposure to these toxins, increasing hepatic portal venous blood flow, and improving liver metabolism [13, 14, 15, 16, 17]. This mechanism is akin to strategies employed in managing refractory HE [17, 18] post‐TIPS using drug treatments, shunt reduction, and smaller or less expandable stents to prevent HE [10, 19, 20, 21, 22]. Currently, TIPS is increasingly utilised worldwide for treating complications of portal hypertension in decompensated cirrhosis [6, 11]. Despite this, HE remains one of the most common and significant complications, limiting the widespread use of TIPS. HE, particularly overt HE, significantly negatively impacts patients' quality of life and survival [23]. Furthermore, with the broad application of potent antiviral therapies in recent years, patients with chronic HBV infection often show reduced intrahepatic inflammation or even reversal of cirrhosis [24]. However, it is essential to note that even in patients undergoing TIPS with concurrent L‐SPSSs embolization, the risk of postoperative HE cannot be entirely eliminated. Therefore, pharmacological prevention of HE remains crucial for these patients. No serious concurrent anterograde embolization‐related complications, such as coil migration causing ectopic embolism, were observed in the L‐S group. One possible explanation is that all joint embolizations were performed prior to TIPS creation. This procedural sequence may contribute to the observed safety and efficacy of the combined approach [19, 25].

In the setting of refractory gastroesophageal variceal haemorrhage, creation of a TIPS to decompress the portal system is often combined with variceal embolization to directly treat the source of haemorrhage and decrease rebleed rates [26]. While coil or plug embolization is performed to interrupt flow into the variceal circuit, the addition of sclerotherapy to permeate through and eliminate the variceal circuit has been suggested as a more efficacious therapy with lower rebleed rates [27, 28, 29]. Controlling the distribution of sclerosants in a high‐flow system that may have large capacitance and multiple inflow vessels; however, may result in incomplete treatment and the potential for non‐target embolization. Systemic embolization is known as a serious complication of the treatment of gastroesophageal varices [30]. There are reports of systemic embolization during antegrade embolization, such as in cerebral, splenic, and coronary arteries. Fortunately, We're more concerned about the rate of ectopic embolization in our study. In our centre, we used coils and/or tissue gel to perform concurrent anterograde embolization. In fact, one patient occurred the coils escaping. Fortunately, the escape coils stalled in the distal part of the target vessel. Meanwhile, no patients had reversed flow in the main portal vein during the antegrade embolization. Because of our interventional therapy, we will place the coils firstly and then microcatheter superselect to the distal end of the coils before injecting the tissue glue. Also, some patients were found to have microsymptomatic pulmonary embolization on postoperative follow‐up review of the contrast‐enhanced abdominal CT scans. Therefore, in the preprocedural assessment for TIPS or variceal eradication candidacy, specific evaluation for history of cerebrovascular accident of unknown aetiology, hypercoagulable states, elevated pulmonary pressures, large liver tumours, or portal vein thrombosis should prompt more thorough cardiopulmonary testing including echocardiography with microbubbles and chest CT with contrast [26].

The study has several limitations. First, being a single centre retrospective analysis, selection bias cannot be entirely ruled out, and larger multicentre studies are necessary to validate these results. Second, there is no unified standard for defining L‐SPSSs, leading to potential subjective measurement errors. However, a recent study suggests that using spontaneous shunt volume or total cross‐sectional area, rather than a single diameter, might be more reasonable due to the commonality of multiple SPSSs in late‐stage chronic liver disease [31]. Third, varying operator experience and incomplete preoperative portal pressure gradient (PPG) measurements limit our ability to assess pre‐ and postoperative PRG changes and the differences between the groups.

In conclusion, TIPS combined with concurrent anterograde embolization of L‐SPSSs offers significant benefits in terms of long‐term survival and reducing the risk of overt HE for cirrhotic patients with portal hypertension and variceal bleeding. Future studies should aim to standardise procedures and validate these results in larger, multicentre cohorts.

## Ethics Statement

All procedures were performed in accordance with the ethical standards of the institutional and/or national research committee and the 1964 Helsinki declaration and its later amendments or comparable ethical standards. The retrospective study was approved by the institutional review board of the participating hospital.

## Consent

The authors have nothing to report.

## Conflicts of Interest

The authors declare no conflicts of interest.

## Data Availability

The data that support the findings of this study are available from the corresponding author upon reasonable request.
